# Primary malignant melanoma of prostate: a case report

**DOI:** 10.11604/pamj.2023.44.56.37632

**Published:** 2023-01-27

**Authors:** Pirzada Faisal Masood, Umesh Sharma, Rajeev Sood, Arvind Ahuja

**Affiliations:** 1 Department of Urology and Renal Transplant, Atal Bihari Vajpayee Institute of Medical Sciences and Dr. Ram Manohar Lohia Hospital, New Delhi, India,; 2 Department of Pathology, Atal Bihari Vajpayee Institute of Medical Sciences and Dr. Ram Manohar Lohia Hospital, New Delhi, India

**Keywords:** Melanoma, prostate, metastasis, case report

## Abstract

While primary genitourinary melanomas account for less than 1% of all melanoma cases, prostatic melanoma is extremely uncommon. These patients are challenging to identify, with a dismal prognosis. We reported a 52-year-old male patient who presented with lower urinary tract symptoms in the last one and a half months. The patient underwent Holmium laser enucleation of the prostate, and the histopathology result of the resected specimen showed prostatic melanoma. Investigations for systemic melanoma evaluation were negative, and the patient underwent radical cystoprostatectomy, urethrectomy, and bilateral lymph node dissection. The patient refused chemotherapy, developed lung metastasis shortly after surgery at three months, and succumbed to the metastatic disease with overall survival of 6 months. In conclusion, primary malignant melanoma of the prostate is a very rare disease. The most logical therapeutic strategy is aggressive surgical resection, followed immediately by adjuvant therapy.

## Introduction

Malignant melanoma rarely involves the genitourinary tract (less than 1%). The most commonly affected genitourinary organ is the urinary bladder, followed by the adrenal glands, kidney, female urethra, and penis [1]. Melanoma of prostatic origin is sporadic, with most cases discovered incidentally after surgical resection of the prostate, and their no ample literature regarding its management [1,2]. Prostatic melanoma represents an exquisite challenge for the urologist as its symptoms are non-specific and mimic benign prostatic enlargement [3,4]. We herein report a case of a 52-year-old male patient who presented with lower urinary tract symptoms and was diagnosed with prostatic melanoma histopathologically.

## Patient and observation

**Patient information:** a 52-year-old male presented to us with progressively increasing lower urinary tract symptoms (LUTS) over the last one and a half months. The patient was undergone Holmium laser enucleation of the prostate (HoLEP) 2 months ago for the same symptoms. The biopsy result of the resected mass was suggestive of prostate melanoma, with immunohistochemistry positive for S-100, HMB-45, and Melan A. The Patient was hypertensive and diabetic, with good control on regular medication for the last eight years. There was no significant family history of malignancy.

**Clinical findings:** ocular and body skin surface examinations were normal. On digital rectal examination (DRE), the prostate was firm in consistency, grade 3 in size, and appeared fixed to lateral pelvic wall without any detectable nodule.

**Figure 1:**
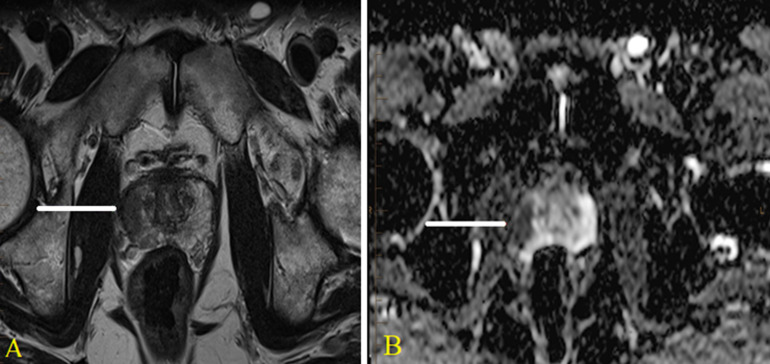
magnetic resonance imaging of the prostate showing - A) T2-weighted indicating lesion in the peripheral zone (white line); B) apparent diffusion coefficient (ADC) indicating focal mild to moderate hypointensity in the peripheral zone (white line)

**Figure 2:**
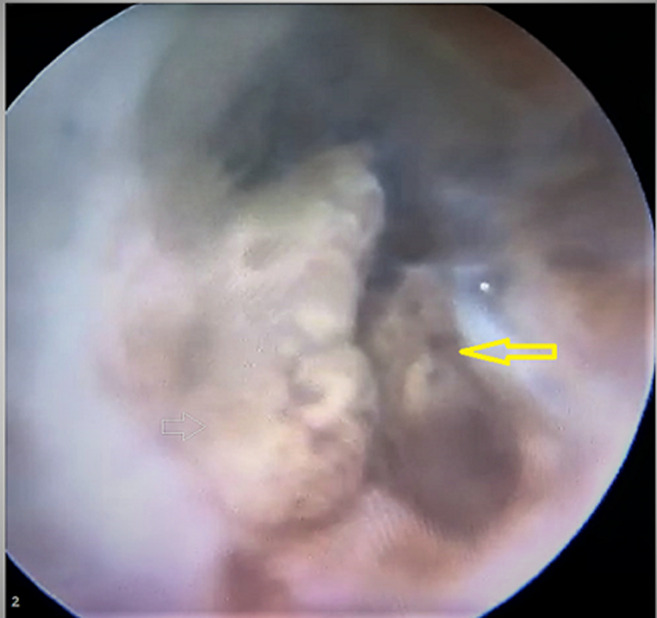
cystoscopy photo of pigmented lesion (arrow)

**Diagnostic assessment:** blood investigation showed haemoglobin 12 g/dl, blood urea nitrogen (BUN) 24 mg/dL, creatinine 0.4 mg/dL, and prostate-specific antigen (PSA) 0.2 ng/dL. Multiparametric Magnetic resonance imaging (MRI) of the abdomen and pelvis revealed 49cc of the prostate, with a lesion in the right peripheral zone. The lesion was equivocal with negative diffusion contrast enhancement (DCE) corresponding to a Prostate Imaging-Reporting and Data System score of three (PIRAD III) (Figure 1 (A,B)). On cystoscopy, the urethra had a 1 x 0.5 cm melanomatous patch in the proximal bulbar urethral region and nodular pigmented growths in the prostatic fossa (Figure 2). Ureteroscopy and upper and lower gastrointestinal endoscopy were done, and all were normal. Contrast-enhanced computed tomography (CECT) of the abdomen, pelvis, and chest were normal. 18F-fluorodeoxyglucose (FDG) positron emission tomography (PET CT) showed high uptake in the right lobe of the prostate (SUV max 32) and a few FDG avid sub centimetric pelvic nodes (SUVmax-8) and no distant metastasis.

**Figure 3:**
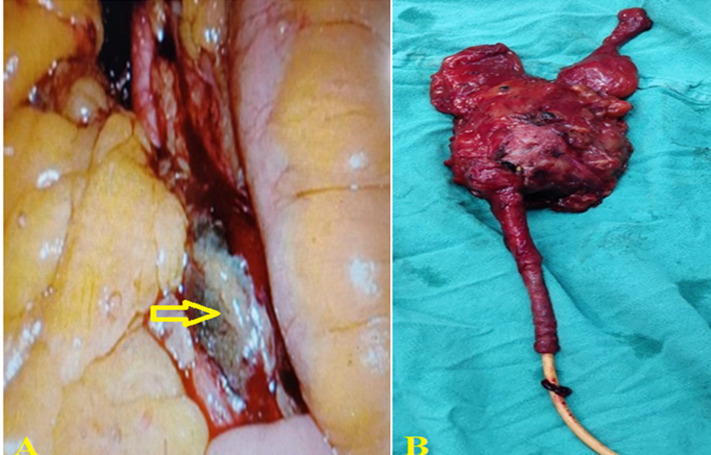
A) enlarged blue-black pelvic lymph nodes (arrow); B) cystoprostatectomy with urethra specimen

**Therapeutic interventions:** the patient was discharged from the hospital as he did not give consent for surgery but was admitted again two weeks later after counseling. The patient underwent laparoscopic radical cystoprostatectomy with extended pelvic lymph node dissection, which, to our surprise, showed enlarged blue-black pelvic lymph nodes, which were not there initially in imaging (Figure 3A). Urethrectomy was done perineally, and the whole specimen was delivered into the abdomen in toto (Figure 3B). A small midline incision was given, the specimen retrieved, and an ileal conduit was made.

**Figure 4:**
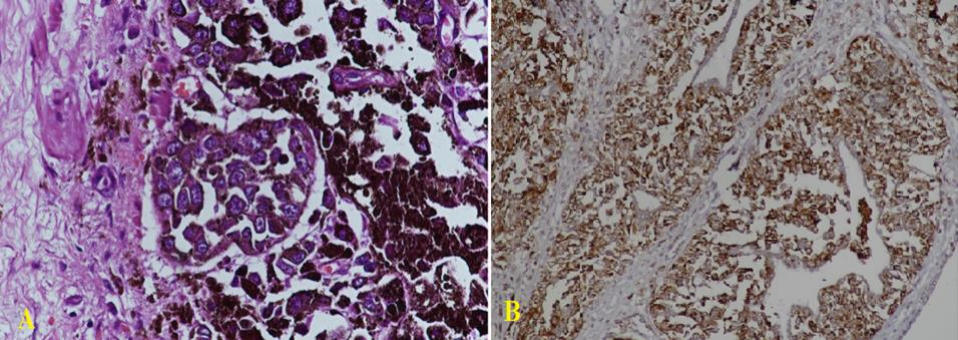
histopathological photos showing - A) round nuclei tumor cells with prominent nucleoli and moderate cytoplasm, both intracellular and extracellular deposit of melanin pigment is seen [H&E,400X]; B) tumour cells with cytoplasmic positivity for HMB-45 [IHC,200X]

**Follow-up and outcomes:** the patient was discharged from the hospital on five postoperative days. Conduit output was around 2 litres per 24 hours. A pathological assessment on gross examination revealed a 4 x 2 cm growth involving the prostate with an extension into the bladder. The growth was infiltrating up to the external surface of the prostate, and the overlying bladder wall was normal. On microscopy, sections from the prostate showed prostate glands with tumor infiltration and tumor cells arranged in nests and sheets. The cells were polygonal in shape with spindle nuclei containing intracellular and extracellular melanin. 6/11 lymph nodes showed metastasis. Immunohistochemistry (IHC) was positive for human melanoma black 45 (HMB-45) and melan-A (Figure 4 A,B). The patient refused chemotherapy and developed pulmonary metastasis at three months. At four months, the patient succumbed to the disease from a lower respiratory tract infection, with overall survival of 6 months.

**Patient perspective:** during treatment, the patient and his family were satisfied with the level of care provided to him.

**Informed consent:** written informed consent was obtained from the patient family for participation in our study.

## Discussion

Primary prostatic melanoma is rare and is labelled only when metastasis from other sites has been ruled out [5]. Out of prostatic melanomas, primary cases represent only a minority. Approximately three out of four prostatic melanomas are metastases from another site [6,7]. Patients with primary prostatic melanoma are usually younger (median, 51.5 years) than those with metastatic melanoma in the prostate (median, 66 years). Most primary cases present with lower urinary tract symptoms (LUTS), while 75% of metastatic prostatic melanoma have LUTS. This could be in metastatic melanoma; the lesion may be small and peripheral and may not be impinging on the urethra [6]. The most likely origin of primary prostatic melanoma is the prostatic urothelium. Metastasis from other sites is usually located peripherally or in the stroma. As LUTS is the commonest presentation, it is the commonest cause of significant diagnostic delay in adults and even improper therapy, as LUTS can easily be confused with benign prostatic hyperplasia, as happened in our case [4]. The result is a diagnostic delay, which adds further dismal prognosis to an already poor prognostic condition. Prostate melanoma is primarily diagnosed on transurethral resection (TUR) chips. On urethroscopy, blackish-brown tissue is a classical melanoma finding, as was seen in our case [8,9]. A staging work-up should be done to determine the disease's extent and treatment accordingly. In our case, all systemic investigations were normal.

In general, radical (cysto) prostatectomy with lymphadenectomy and possibly adjuvant chemotherapy or immunotherapy is the treatment of choice for primary prostatic melanomas. On the other hand, treatment of melanoma metastases to the prostate depends on the overall clinical status and can consist of transurethral resection of the prostate (TURP) or systemic chemo-immunotherapy [2,9]. Involvement of the urethra needs urethrectomy, as was done in our case. On histopathology, most cases are pigmented, with no difference between primary and metastatic cases. They should be differentiated from melanosis by IHC. On IHC, HMB-45 is positive in almost 100% of primary and secondary prostatic melanoma. The same has been reported with melan-A. S-100 expression has been reported in 67% of primary and 100% of secondary prostatic melanoma [6,10]. Systemic therapies include chemotherapy, targeted therapy, and immunotherapy. Dacarbazine, Vinblastine, and Cisplatin are the chemotherapeutic agents commonly used. Immunotherapeutic agents like Ipilimumab and Nivolumab are widely used in melanoma, either alone or in combination with other drugs [11]. In our case, the patient refused any adjuvant therapy and developed a lung metastasis shortly.

The prognosis of primary prostatic melanoma is not that bad, provided timely intervention is done [12]. The treatment window can be lost if there is any delay, as was evident in our case on laparoscopy (the overall survival in our case was six months). Survival analysis has revealed a longer overall survival in patients with primary prostatic melanomas. In Caputo *et al*. study, among the 7 cases of primary prostatic melanoma with at least one year of follow-up, two survived more than five years, while the remaining five died after one year [6]. Prostatic metastases from melanoma, on the other hand, carry a dismal prognosis, with a median survival of only three months [6].

## Conclusion

Primary malignant melanoma of the prostate is a very rare disease. After diagnosing prostatic melanoma, the most important clinical distinction to make is between primary and secondary melanoma, as the latter carries a dismal prognosis while the former can be curable by surgery if localized. As a result, it is critical to conduct a thorough search for a faraway melanoma. There should be no delay in instituting the treatment, as the therapeutic window can be lost. The most logical therapeutic strategy is aggressive surgical resection, followed immediately by adjuvant therapy.
